# Resonant Cavity Effect for Spectrally Tunable and Efficient Narrowband Perovskite Photodetectors

**DOI:** 10.1021/acsphotonics.4c01942

**Published:** 2025-07-21

**Authors:** Zher Ying Ooi, Shenyu Nie, Guadalupe Vega, May Ching Lai, Alberto Jiménez-Solano, Chieh-Szu Huang, Hao Wang, Tianjun Liu, Krzysztof Gałkowski, Michał P. Nowak, Piotr Nyga, Qixiang Cheng, Caterina Ducati, Sol Carretero-Palacios, Simon Kahmann, Samuel D. Stranks, Miguel Anaya

**Affiliations:** † Department of Chemical Engineering and Biotechnology, 2152 University of Cambridge, Cambridge CB2 1TN, U.K.; ‡ Instituto de Ciencia de Materiales de Sevilla, 16778 Universidad de Sevilla−CSIC, Calle Américo Vespucio 49, Sevilla 41092, Spain; § Departamento de Física, 16735 Universidad de Córdoba, Edificio Einstein (C2), Campus de Rabanales, Córdoba 14071, Spain; ∥ Department of Materials Science and Metallurgy, University of Cambridge, Cambridge CB3 0FS, U.K.; ⊥ Division of Electrical Engineering, Department of Engineering, University of Cambridge, Cambridge CB3 0FA, U.K.; # Cavendish Laboratory, University of Cambridge, Cambridge CB3 0HE, U.K.; ∇ Department of Experimental Physics, Faculty of Fundamental Problems of Technology, Wroclaw University of Science and Technology, Wroclaw 50-370, Poland; ○ Institute of Optoelectronics, 69698 Military University of Technology, Warsaw 00-908, Poland; ◆ Instituto de Ciencia de Materiales de Madrid, ICMM-CSIC, Madrid 28049, Spain; ¶ Institute of Physics, Chemnitz University of Technology, Chemnitz 09107, Germany

**Keywords:** halide perovskite, narrowband photodetector, resonant cavity, angular tunability

## Abstract

Narrowband photodetectors with precise spectral control offer significant potential for applications such as color imaging and machine vision. However, existing demonstrations have encountered challenges due to restricted absorption, the need for additional filters, or the inclusion of thick absorbing layers to facilitate charge collection filtering mechanisms. These constraints have resulted in suboptimal detectivity, inadequate color control, or slow response. Here, we exploit cavity resonance enhancement to demonstrate a highly spectral selective and robust perovskite photodetector, showing 2.4-fold EQE enhancement at the main narrowband peak with respect to a broadband photodetector counterpart of the same perovskite thickness. This device architecture achieves peak external quantum efficiency of 80%, responsivity of 0.41 A W^–1^, and detectivity of 3.7 × 10^11^ Jones at the main narrowband peak, with a secondary signal below 450 nm that can be mitigated with advanced photonic crystal as proposed. Additionally, the resonant cavity-enhanced photodetector offers a rapid switching of 0.9 μs and low noise of 0.57 pW Hz^–1/2^. Our demonstration shows precise tuning of the main narrowband photodetection characteristics across a 100 nm spectral range by simply varying the thickness of the perovskite layer, ensuring device efficiency and stability across the wavelength region around 560 to 660 nm, where most perovskite devices suffer from degradation due to halide segregation. This work demonstrates the practical integration of resonant cavity enhancement in perovskite photodetectors and paves the way for high-performance optical sensing, multispectral imaging, and wavelength-selective photonic devices.

## Graphical abstract

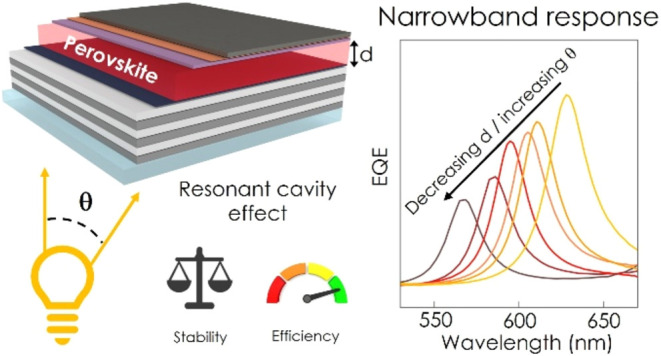

## Introduction

Photodetectors serve as components that transform optical signals into electrical signals for processing by electronic circuits.^1^ They have found widespread implementation across various domains, including optical communication, autonomous driving, image sensing, environmental monitoring, and fluorescence imaging, among others.^2,3^ Spectral selectivity across the full color spectrum is essential for color-specific applications such as machine vision,^4^ multispectral sensing,^5^ and hazard detection.^6^ In numerous applications, it is essential to achieve required signal detection accuracy by reducing background noise and minimizing cross-talk.^3,7^ To achieve narrowband and controlled spectral response, conventional technology uses broadband monocrystalline semiconductors such as silicon and InGaAs, along with accompanying optics as filters.^8^ However, this approach introduces spatial resolution limitations, color artifacts due to imperfect filtering and intensity loss caused by the filters partially blocking incoming light of desired wavelength, which reduces the photodetection quantum efficiency.^8,9^ Recently, selective types of organic materials with narrow absorption spectra have enabled inherent narrowband photodetection, but their spectral control is constrained by the absorption nature of organic materials which typically have a spectral full width at half-maximum (FWHM) above 100 nm.^10^ Moreover, charge collection narrowing (CCN) approach has gained popularity among emerging absorbing materials, including polymers and organics.^4,11^ In this approach, spectral selectivity is achieved by modulating the charge collection process, in which the thickness of the absorption layer is adjusted to match the spectrally dependent penetration depth of light.^12^ However, narrowband photodetection through CCN suffers from low external quantum efficiency (EQE) and slow response time due to a very thick absorbing layer.^4,11^ Alternatively, the implementation of cavity resonance within photodetectors was first demonstrated in III–V semiconductor photodetectors and offers numerous benefits. This approach enables the use of thin active layers, leading to high quantum efficiencies and rapid response times, as well as allowing for adjustable FWHM, precise narrowband wavelength selectivity and sensitivity to incident light angles.^13,14^ However, fabricating III–V semiconductor photodetectors involves high costs due to intricate manufacturing processes.

Halide perovskites have arisen as attractive light harvesting materials due to their facile bandgap tunability across the ultraviolet to the near-infrared spectral region, as well as solution processability and large absorption coefficient, charge carrier mobility, and collection efficiency.^8,15^ Perovskite devices have shown great photodetection performance under zero bias that enables self-powered operation, reaching an EQE of 90%, responsivity of 0.41 A W^–1^ at 630 nm, and response time of 2 μs.^16^ This performance rivals that of commercial silicon photodiodes with a similar active area.^17^ However, the broadband nature of absorption in these perovskites limits their use for narrowband photodetection applications. To date, narrowband photodetection using a perovskite as the absorber material has been demonstrated with external filtering and CCN. Reported performance in perovskite-based narrowband photodetectors using external filtering shows an EQE of 50%, an FWHM of 50 nm, and a response time of 200 μs,^18^ whereas photodetectors utilizing CCN achieve an EQE of 10%, an FWHM of 30 nm, and a response time of 10 μs through CCN.^12^ More demonstrations of external filtering^9^ and CCN^6,19–23^ in perovskite photodetectors are detailed in Table S2.

Existing methods, including external filtering and CCN rely heavily on compositional engineering of halide perovskites for spectral response tuning. While halide perovskites offer flexibility in bandgap tunability through composition variation, their device performance across the entire spectrum is not uniformly high. For example, high bromide-to-iodide ratios can lead to phase segregation, which adversely affects efficiency and stability, resulting in a shortage of effective perovskite devices for the 560 to 660 nm range.^24,25^ Such gaps in the spectrum limit their application capabilities and complicate system design. Therefore, achieving fine-tuning of the narrowband photodetection spectrum without intricate compositional adjustments, particularly in the phase-segregation-prone regions, is essential but remains challenging with currently reported techniques. In contrast, an unexplored alternative in the perovskite photodetection field is the use of a resonant cavity, which has shown improved performance and flexibility in III–V semiconductors, silicon, graphene, organic, and colloidal quantum dots photodetectors.^26–29^ This approach enhances efficiency by localizing the electric field within the absorbing layer within a narrow spectral range of around an FWHM of 30 nm,^27^ thereby allowing a reduced absorber thickness down to 50 nm thick^29^ and shorter charge carrier collection time than comparable controls without resonance effects. More details can be found in Supporting Note 2.

In this study, we report high-performance narrowband photodetectors with an operational window tunable between 560 and 660 nm by using a stable perovskite composition and adjusting the layer thickness within the resonant cavity, covering a wavelength range that is inaccessible through perovskite compositional engineering. Our approach features a resonant cavity-enhanced perovskite photodetector that incorporates a combination of metal and dielectric mirrors. This approach demonstrates a main narrow spectral operation window with an FWHM of 38 nm, where light confinement boosts absorption and enables the use of perovskite layers of tens of nanometers. This thin character, in turn, results in better charge carrier collection for quantum efficiency and responsivity up to 80% and 0.41 A W^–1^, respectively, which represents a 2.4-fold enhancement in performance with respect to a broadband photodetector counterpart of the same thickness. The photonic character of the device is confirmed by the electrical signal in the UV-blue region below 450 nm, which can be mitigated by employing alternative, advanced configurations as calculated. The incorporation of hybrid metal-dielectric mirrors into perovskite photodetectors demonstrates minimal modification to conventional device architecture, offering flexible and effective spectral tuning across the visible region, with potential for adaptation to other absorbers, enabling applications like high-resolution spectral detection, such as high-precision color imaging, wavelength-selective sensing, and advanced optical communication systems.

### Resonant-Cavity-Enhanced Perovskite Photodetector

We employ a standard perovskite solar cell architecture for our photodetector design, consisting of indium tin oxide (ITO)/[2-(3,6-Dimethoxy-9H-carbazol-9-yl)­ethyl]­phosphonic acid (MeO-2PACz)/ FA_0.8_Cs_0.2_Pb­(I_0.6_Br_0.4_)_3_ perovskite/carbon-60 (C_60_)/bathocuproine (BCP)/silver (Ag), as illustrated in Figure 1a. The thicknesses of each layer are subsequently tuned for optimum optical and tunable device properties. In this configuration, ITO and Ag serve as electrodes, MeO-2PACz is a self-assembled monolayer functioning as the hole-transport layer, while C_60_ and BCP are utilized as the electron-transport layer and interface buffer layer, respectively. Beyond acting as contact to extract electrons, Ag acts as a mirror for the optical cavity, which is completed by the multilayer structure beneath the ITO functioning as a photonic crystal.^30^

**Figure 1:**
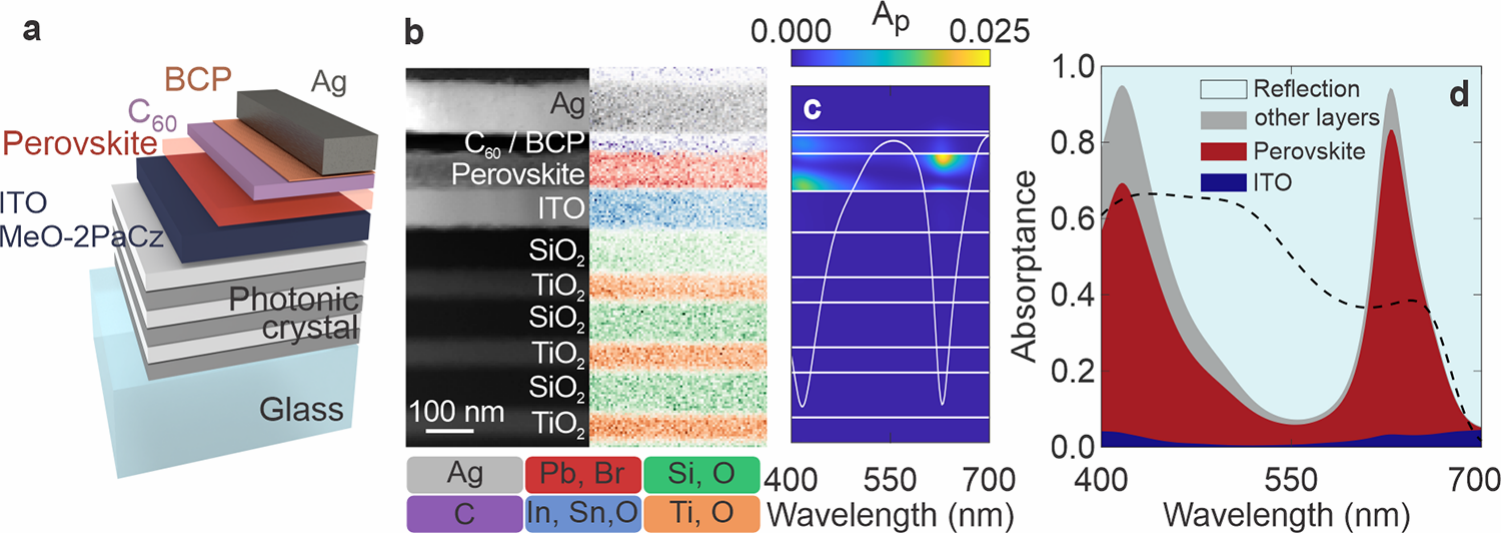
Resonant cavity-enhanced perovskite photodetector. (a) Schematic of the resonant cavity photodetector. Photonic crystals consist of three alternating TiO_2_ and SiO_2_ bilayers. (b) Cross-sectional HAADF-STEM image (left), and energy-dispersive X-ray (EDX) chemical map (right) of the resonant cavity photodetector. (c) Simulated spectral absorption across the cross-section of the device stack. The white line shows simulated reflectance of the structure. (d) Simulated ratio of integrated absorption within layers of the structure and the resulting reflection from the whole device stack. Absorption within TiO_2_, SiO_2_, C_60_, BCP, and Ag is detailed in Figure S6. Dashed line shows the perovskite absorptance in a reference device structure.

We selected SiO_2_ and TiO_2_ as the building blocks for the photonic crystal due to their physical and chemical robustness and suitability to be deposited on glass.^30^ These materials are transparent in the optical range and have refractive indices closely matched to those of ITO and perovskite (Figure S1). This strategy takes advantage of the inherent photodetector architecture, adding an additional unit cell to the photonic crystal. To optimize the photodetector device structure within the cavity, a transfer matrix model was employed.^31^ Figure S1 provides the optical constants of the materials used in the model. The optical constants of the (FA_0.8_Cs_0.2_Pb­(I_0.6_Br_0.4_)_3_) perovskite layer were derived from fitting its transmittance and reflectance spectra at different angles (see the Methods section). Initial simulations focused on a SiO_2_/TiO_2_ reflector with quarter-wavelength thickness and the transport layers, as shown in Figures S2 and S3, respectively, followed by the incorporation of a conventional broadband photodetector device structure, which is also the reference photodetector for comparison (see the standalone device architecture in Figure S4). To assess the best parameters of the resonant cavity photodetector structure, we conducted simulations varying parameters such as the number of photonic crystal SiO_2_/TiO_2_ bilayers (Figure S5) and perovskite thickness (Figure S6). The findings indicate that a resonant cavity design with a resonance wavelength above 550 nm, composed of three photonic crystal bilayers and supporting a single-cavity mode, already demonstrates strong resonance within the perovskite layer. The thicknesses of the various layers were then fine-tuned to achieve a sharp resonance and strong absorption within the perovskite layer. The ITO thickness was varied between 50 and 160 nm as variations within this range were found to have no significant impact on device performance. The perovskite layer was optimized within a range of 30 and 100 nm to effectively shift the cavity resonance. These optimizations led to the proposal of a resonant cavity photodetector structure that not only demonstrates strong resonance but is also simple to fabricate, requiring minimal photonic crystal bilayers.

The experimental realization of the designed resonant cavity perovskite photodetector is displayed in Figure 1b, where cross-sectional high-angle annular dark-field scanning transmission electron microscopy (HAADF-STEM) reveals the structure consisting of three bilayers of SiO_2_ (96 nm) and TiO_2_ (56 nm), along with ITO (90 nm), MeO-2PACz (∼1 nm), perovskite (82 nm), C_60_ (40 nm), BCP (8 nm), and Ag (100 nm). Since the organic components have similar atomic compositions and densities, the thicknesses of C_60_ and BCP layers were confirmed by thickness measurement using an atomic force microscope (AFM). The HAADF-STEM analysis carried out on a 1 × 0.5 μm section confirms the high uniformity of the e-beam deposited SiO_2_/TiO_2_ photonic crystal and sputtered ITO layers, which contribute to the device’s high optical quality. The homogeneous photonic crystal and ITO layers facilitate the high-quality growth of perovskite film, akin to its typical growth on an ITO layer on glass substrates, resulting in a high-performing device as we will discuss later. The perovskite layer itself is also uniform and smooth, as evidenced by AFM, which shows an average roughness of 4.44 nm (Figure S7). Elemental mapping by energy-dispersive X-ray (EDX) further confirms the well-defined material interfaces, with a pinhole-free perovskite layer that is essential for the photodetector’s operational capability. This analysis demonstrates that the photodetector, fabricated on the underlying photonic crystal multilayers, exhibits no negative impact and features uniformly high-quality layers.

Simulations of the resonant cavity perovskite photodetector, as shown in Figure 1c,d, that closely align with the experimental cross-sectional images, shows spectrally maximized effective absorption at the resonance centered at 630 nm, within the typical wavelength range for standard pure red in commercial LEDs.^32^ The narrowband absorption exhibits an FWHM of 40 nm, with 83.4% of light absorbed by the 82 nm thick perovskite. Simultaneously, as shown in Figure S6, the parasitic absorption is minimized as compared to the reference structure, which is crucial for optimizing EQE values in the narrowband photodetector. In contrast, the reference photodetector without the integrated photonic crystal shows a broadband absorption profile, with light absorption gradually increasing with increasing perovskite thickness, achieving around 80% absorption above 280 nm thick perovskite. The simulation demonstrates that light absorption in the perovskite layer is enhanced by more than a factor of 2, as determined by comparing the absorption of the resonant cavity structure to that of the reference photodetector (see Figure S6). Therefore, such a design not only narrows the spectral response but also substantially reduces the perovskite thickness, which enables more efficient charge extraction, as discussed later.

### Tunable Resonance Spectrum

As shown in Figure 2a, the reflectance curve of the resonant cavity perovskite photodetector with an 82 nm thick perovskite layer exhibits a sharp resonance with an FWHM of 38 nm and a minimum reflectance of 4.7% at 632 nm, enabling high selectivity at this wavelength. The reflectance curve is strongly anticorrelated with the EQE curve, as shown in Figure 2b, achieving a peak EQE of 79.8% at 632 nm, also with a narrowband FWHM of 38 nm. The device operates in a self-powered 0 V bias unless otherwise specified. For comparison, we examined the reference photodetector without a photonic crystal (see Figure S4), also featuring an 82 nm thick perovskite absorber. The reference photodetector shows a broadband EQE curve spanning 310 to 690 nm, achieving 32.8% at 632 nm. As shown in Figure 2c, the EQE enhancement, calculated as the ratio between the EQE of the resonant cavity and that of the reference photodetector, demonstrates a 2.4-fold enhancement at 632 nm, closely aligning with the simulation results. The high reflectance of 84.5% at off-resonance wavelengths suggests strong filtering, leading to low EQE signals outside the resonance peak. The EQE enhancement outside the resonance wavelength is nearly zero, confirming strong narrowband selectivity by the resonant cavity, which is crucial for avoiding artifacts in practical applications. As our current photonic crystal does not suppress the photodetection response below 450 nm, as detailed in Supporting Note 1, more advanced photonic architectures can be implemented to mitigate this secondary signal, as shown in Figure S8.

**Figure 2:**
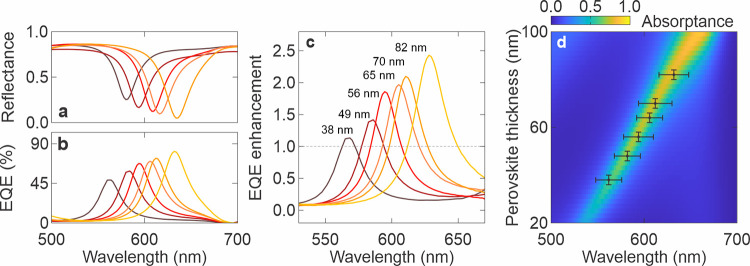
Tunability of cavity resonance with perovskite thickness. (a) Measured reflectance and (b) EQE of resonant cavity photodetectors with varying perovskite thickness from 38 nm (brown) to 82 nm (yellow). (c) EQE enhancement defined as the ratio of EQE of cavity photodetector to that of the reference photodetector with the same perovskite thickness as labeled in the graph. (d) Simulation depicting the shifting of cavity resonance with varying perovskite thickness. Measured EQE narrowband peak wavelengths were mapped on the graph.

The photonic character of our resonant cavity photodetector enables fine-tuning of the narrowband operational window by simply adjusting the thickness of the perovskite layer, as illustrated in Figure 2d. To validate the simulation findings, we fabricated a series of resonant cavity photodetectors with active perovskite thicknesses of 38, 49, 56, 65, 70, and 82 nm. The perovskite thickness was achieved by diluting the perovskite precursor concentration to 0.17, 0.20, 0.22, 0.25, 0.27, and 0.30 M, respectively. As shown in Figure 2a,b, decreasing the perovskite thickness from 82 to 38 nm causes a blue shift in the resonance, from 632 to 562 nm, observed in both the reflectance and the corresponding narrowband EQE peak, which is attributed to the reduction in cavity length. Additionally, the intensity of narrowband EQE peaks gradually decreases as the resonance wavelength decreases, mirroring the decreasing resonance strength seen in the reflectance curve. Nevertheless, when evaluating the EQE ratio between the resonant cavity and reference photodetectors with their corresponding perovskite thickness, there is significant EQE enhancement in all cases ranging from 2.4-fold to 1.5-fold as the cavity resonance shifts from 632 to 562 nm, respectively, with decreasing perovskite thickness (see Figure 2c), which matches the trend of the calculated resonance strength (Figure 2d). The calculations shown in Figure 2d demonstrate the enormous potential of our self-powered, resonant cavity photodetector to achieve a narrowband response over an operational window from 560 to 660 nm.

### Sensitivity to Incident Light Angles

Figure 3a,b demonstrates the angle dependency observed in the resonant cavity photodetector with an 82 nm thick perovskite absorber. As the specular reflectance is measured at angles ranging from 8 to 60°, the wavelength of the reflectance minimum shifts from 630 to 574 nm, while the reflectance minimum value increases from 5.3 to 38.9%. This indicates the strongest resonance occurs at normal incidence, the angle for which we optimized the device through optical design. The narrowband EQE spectra again show an anticorrelation with the reflectance curve with the EQE peak shifting from 632 nm at 0° (630 nm at 8°) to 574 nm at 60°. The EQE presented here is normalized to that at normal incidence. As the angle increases, both reflectance and narrowband EQE exhibit slight broadening, with FWHM increasing from 38 nm at 0° to 48 nm at 60°, which still maintains the narrowband characteristics. Thus, varying the incidence angle between 0° and 60° results in a parametrizable resonance shift up to 60 nm in narrowband response, which holds potential for applications requiring angular optical alignment. Further measurements, as shown in Figure S9 for resonant cavity photodetectors with 56, 65, and 70 nm thick perovskite absorbers, reveal a similar trend, confirming the consistency of these angle-dependent observations. This angle dependency is typical for resonant cavity structures due to changes in the optical path, the effective refractive index of the medium, and interference at various angles of incidence.^29^

**Figure 3:**
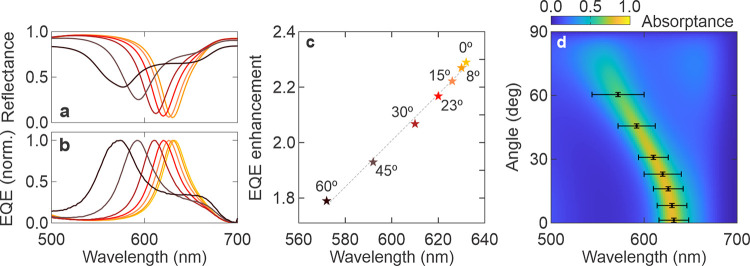
Angle-dependent cavity resonance. (a) Measured reflectance (from 8 to 60°) and (b) EQE of resonant cavity photodetectors consisting of 82 nm thick perovskite layer with increasing incident illumination angle from 0° (yellow) to 60° (brown). (c) EQE enhancement defined as the ratio of EQE of the cavity photodetector to that of the reference photodetector with the same perovskite thickness, which are both 82 nm. The dashed line shows a linear fitting across the points. (d) Simulation depicting the shifting of cavity resonance with illumination angle for a resonant cavity photodetector with 82 nm thick perovskite. Measured EQE narrowband peak wavelengths were mapped on the graph.

Angle-dependent EQE measurements were also conducted for the reference photodetector, allowing for the calculation of the angle-dependent EQE enhancement shown in Figure 3c. As the angle increases from 0 to 60°, the EQE enhancement decreases from 2.3-fold (a slightly different value than in Figure 2 due to batch-to-batch variation) to 1.8-fold, consistent with the reflectance curve where resonance strength decreases with increasing angle. Angular-dependent simulations, shown in Figure 3d and overlaid with the experimental EQE peaks, reveal that the experimental results align with the simulated absorption peaks within the perovskite layer across the 0 to 60° range. The pronounced decrease in EQE enhancement with increasing angle, which closely resembles a linear decline, offers effective control over angular sensitivity with simple calibration. Additionally, for scenarios where signals come from a specific direction, such as in optical communications and time-of-flight sensing, this characteristic helps filter out background noise from wide angles, thereby enhancing accuracy, reducing cross-talk, and minimizing artifacts.

### Perovskite Photodetector Performance

As shown in Figure 4a, the champion resonant cavity perovskite photodetector with an 82 nm thick perovskite absorber layer achieves a responsivity of 0.41 A W^–1^ at 632 nm and a specific detectivity of 3.7 × 10^11^ Jones considering the responsivity at 0 V bias, device area, and estimated noise current detailed in the following paragraph. We note that both our ITO and photonic crystal substrates were recycled and reused for up to five iterations. The devices fabricated from recycled substrates exhibited negligible performance degradation, as evidenced by consistent EQE measurements throughout the recycling process, enabling effective device optimization using a limited number of substrates (Figure S10). The reference perovskite photodetector with an 82 nm thick perovskite absorber shows responsivity of 0.17 A W^–1^ and specific detectivity of 1.6 × 10^11^ Jones at 632 nm. Noise equivalent power (NEP) of the champion resonant cavity photodetector with an 82 nm thick perovskite, calculated from the maximum specific detectivity across the spectra, is 0.57 pW Hz^–1/2^, while that of the reference photodetector is 1.32 pW Hz^–1/2^. The enhancement in responsivity, specific detectivity, and NEP is 2.4-fold, similar to the observations in EQE spectra, highlighting the importance of photonics in improving the photodetector’s metric.

**Figure 4:**
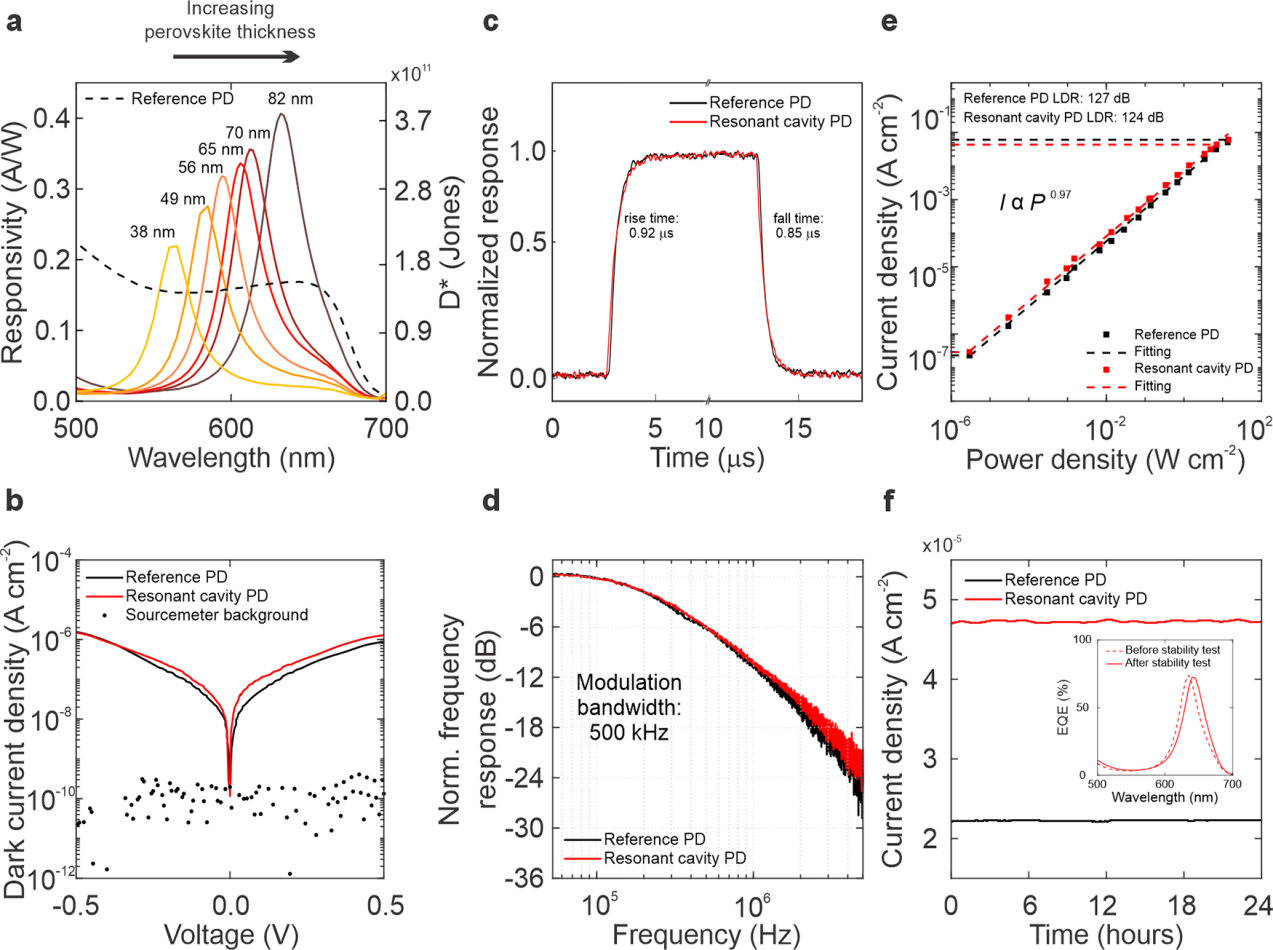
Narrowband perovskite photodetector performance. (a) Responsivity and specific detectivity of resonant cavity photodetectors across increasing perovskite thickness. Responsivity and specific detectivity of reference photodetector with 82 nm thick perovskite in the dashed line. All of the following including both reference and resonant cavity photodetectors have an 82 nm thick perovskite absorber layer. (b) Current density–voltage curve of reference and resonant cavity photodetectors. Source meter noise was shown as scattered dots. (c) Transient response of reference and resonant enhanced cavity perovskite photodetectors. The rise and fall times were calculated from the time for the signal to change from 10 to 90% of the peak response. (d) Frequency response of reference and resonant cavity perovskite photodetectors. The modulation bandwidth is defined as the frequency range over which the optical power is reduced to half of its maximum. (e) Linear dynamic range (LDR) of reference and resonant cavity perovskite photodetectors. The slope of the log–log curve fitting is α = 0.97, which is close to unity, showing an accurate linear fitting. Dotted horizontal lines show maximum and minimum current density points for LDR calculation. (f) Photocurrent stability of unencapsulated photodetectors measured in air under 0 V bias. The photodetectors were illuminated with a lamp emitting at a wavelength of 635 nm and a power intensity of 3 mW cm^–2^. The small fluctuations in the photocurrent signals could potentially be due to fluctuations in lamp intensity. Inset: EQE comparison of fresh device and after 24 h of exposure in ambient air. The small red shift in the EQE spectra after 24 h of exposure for both illuminated and dark pixels, likely due to phase segregation in mixed-halide perovskites photovoltaics.^33^

When the perovskite thickness decreases from 82 to 38 nm, the responsivity narrowband peak blue shifts from 632 to 562 nm, and the responsivity decreases from 0.41 to 0.22 A W^–1^ due to the weakening of resonance strength, showing a similar trend of narrowband response as observed in the EQE spectra in Figure 2b. This follows with a decrease in specific detectivity from 3.7 × 10^11^ Jones to 2.0 × 10^11^ Jones, followed by an increase in NEP from 0.57 to 1.06 pW Hz^–1/2^. As a result of the effective incorporation of resonant cavity enhancement into our perovskite device, the EQE, responsivity, and specific detectivity of our resonant cavity-enhanced perovskite photodetector, across the tunable operational window of 632 to 562 nm, surpass those of narrowband perovskite detectors realized through various methods (Figure S11 and Table S1), as well as broadband perovskite photodetectors (Table S2). Additionally, our resonant cavity-enhanced perovskite photodetector shows competitive specific detectivity and NEP with reported visible range (300–700 nm) photodetectors from other absorber materials, as detailed in Supporting Note 2.

We focus now on the reference and the resonant cavity perovskite photodetectors, both with a perovskite thickness of 82 nm, to further examine the effect of the resonant cavity on the photodetector’s performance. From the current–voltage curve in Figure 4b, the extracted dark current density of both photodetectors is around 1.8 × 10^–10^ A cm^–2^ at 0 V and 1.7 × 10^–7^ A cm^–2^ at −0.2 V, which is expected, as this metric is not affected by the photonic environment. A low dark current, typically achieved by minimizing leakage current, trap-assisted tunneling, and thermal generation of electron–hole pairs, is desirable because it improves the signal-to-noise and sensitivity of the photodetector.^34^ The calculated shot noise current (see the Methods section) extracted from the dark current measured at −0.2 V is estimated to be around 230 fA Hz^–1/2^ for both photodetectors. Since the experiments were conducted in ambient air, the calculated thermal noise current for both photodetectors at room temperature is 16 fA Hz^–1/2^ (see the Methods section). Considering the contribution from both the shot and thermal noise, the estimated total noise current of both photodetectors is around 250 fA Hz^–1/2^. As the current–voltage curves of the reference and the photonic photodetectors are very similar, the resistance, dark current, and thus the calculated shot noise and thermal noise are maintained, confirming that these metrics are not affected by the photonic character of the devices. We note that dark current values are similar independently of perovskite thickness, although they increase after three months of storage (Figure S12). A noise comparison with photodetectors from various absorber materials is included in Supporting Note 2.

The electrical time response and modulation bandwidth of a photodetector are crucial metrics that limit the overall speed and bandwidth of the device, affecting its ability to accurately capture fast-changing optical signals and determine its suitability for high-speed applications such as optical communication, time-resolved spectroscopy, and real-time imaging systems. As shown in Figure 4c, the rise and fall times of both the reference and resonant cavity photodetectors are 0.92 and 0.85 μs, respectively, showing minimal impact of the photonic character on the device’s electrical performance. The instrument response of the measurement system is validated with a faster-responding silicon photodetector, as shown in Figure S13. As shown in Figure 4d, the modulation bandwidth of both the reference and resonant cavity photodetectors is 500 kHz, also a minimal effect of the photonic character on the photodetection speed. A photodetector’s response speed is related to the charge carrier generation rate and transport speed, which depend on the photodetector’s material, active layer thickness, device area (capacitance and resistance), electrode resistance, and device design.^35^ Previous work on perovskite photodetectors has shown that reducing device area decreases geometric capacitance, minimizing the resistor-capacitor (RC) time constant and significantly increasing modulation bandwidth.^2,36^ High-bandwidth perovskite devices, up to tens of MHz, can be achieved with device areas around 0.01 mm^2^ (which is 1/100 of our device area).^36^ Therefore, when considering photodetectors with comparable active areas, our resonant cavity-enhanced devices demonstrate superior response speeds compared to narrowband perovskite photodetectors using CCN due to minimized thickness which fastens charge carrier collection (see Figure S11). Additionally, they exhibit competitive response rates relative to other narrowband perovskite photodetectors, broadband perovskite photodetectors (Table S2), and narrowband detectors made from different materials (Supporting Note 2).

Linear dynamic range (LDR) is a quantity that demonstrates a photodetector’s ability to maintain linearity across a wide range of light levels and is crucial for applications needing precise and reliable light detection.^37^ By assessing the photo response of our photodetectors across varying light intensities, we observe a wide LDR exceeding 120 dB for both devices, as illustrated in Figure 4e, indicating minimal impact on the photonic character. The achieved LDR is competitive with both high-performing broadband^16,38^ and narrowband^23,39^ perovskite photodetectors. Long operational stability is crucial for practical applications and must be checked to validate the accuracy of measurements. The photocurrent of both the reference and resonant cavity photodetectors shows negligible degradation within 24 h of illumination in ambient air without encapsulation (Figure 4f), demonstrating encouraging stability.

## Discussion

As shown in Figure S11, while narrowband perovskite photodetectors using external filters and CCN often exhibit lower performance compared to broadband photodetectors due to inherent losses, our self-powered resonant cavity perovskite photodetectors show notable improvements in EQE, responsivity, specific detectivity, and noise equivalent power at the main narrowband peak. Additionally, they maintain high performance in terms of time response, modulation bandwidth, noise current, linear dynamic range, and stability when compared to parent broadband reference photodetectors (Table S2). The enhancements in EQE, responsivity, and specific detectivity are attributed to the ability of the cavity to boost the response at the resonance, without compromising noise, sensitivity, and speed. However, our resonant cavity device displays inferior spectral purity compared with other approaches in the literature since the photonic crystal stopband has partial transmission in the 300 to 450 nm range, leading to a secondary spectral response in the photodetector. Our simulations suggest that optimizing the photonic crystal thickness could mitigate this effect (see Figure S8).

One-dimensional photonic crystals offer exceptional versatility by adjusting parameters such as layer thickness, number of layers, and refractive index contrast. Here, we have shown that the main narrowband peak of a photodetector can be tuned across a 100 nm range from 560 to 660 nm just by finely changing the perovskite thickness without altering its composition. Our approach contrasts with reported methods such as external filtering, internal filtering using CCN and tandem devices (Figure S11), which would rely on perovskite compositions with high bromide-to-iodide ratios that are prone to degradation due to unwanted phase formation.^40^ The resonant cavity strategy, therefore, opens new avenues to achieve robust narrowband detection in wavelength ranges crucial for applications such as medical imaging, environmental monitoring, or remote sensing.^41,42^ Indeed, our simulations demonstrate that similar performance can be achieved with other perovskite compositions, potentially extending the operational spectral window over the visible spectrum, including the blue and green regions (Figures S14 and S15). In addition, the flexibility of the design offers potential for further expansion, such as incorporating high-quality solution-processed dense photonic crystals^43,44^ or employing electrically responsive photonic crystals to achieve in situ tunable photodetectors.^45^

## Conclusions

We demonstrate a resonant cavity-enhanced perovskite photodetector that features tunable, angle-dependent, and strong narrowband response capabilities. Through a combination of simulations and experimental optimizations, we identified the optimal resonant cavity configuration, utilizing a perovskite absorbing layer as thin as 82 nm. This configuration achieves a main narrowband photo response with an enhancement factor up to 2.4-fold, yielding an EQE of 80%, a responsivity of 0.41 A W^–1^, and an FWHM of 38 nm. By adjusting the perovskite layer thickness between 82 and 38 nm, the narrowband peak can be tuned from 632 to 562 nm, with all devices showing enhanced photoresponse. The resonant cavity-enhanced perovskite photodetector demonstrates outstanding overall performance, including a rapid response of 0.92 μs (rise) and 0.85 μs (fall), a modulation bandwidth of 500 kHz, low noise of 230 fA Hz^–1/2^, high specific detectivity of up to 3.7 × 10^11^ Jones, a low noise equivalent power of 0.57 pW Hz^–1/2^, and a broad linear detection range exceeding 120 dB. It also offers precise spectral control and directional sensitivity. The fabrication of this device requires only a minimal amount of photonic crystal and a very thin absorbing layer, making it highly efficient and desirable. These photodetectors demonstrate potential for narrowband photodetection applications where fast switching, high detectivity, precise spectral control, and angular control are crucial, such as in machine vision, multispectral sensing, and hazard detection.

## Materials and Methods

### Optical Simulation

The simulation of reference and resonant cavity structures are based on the transfer matrix method,^31^ by inputting refractive index of each material as shown in Figure S1. The absorption within each layer is calculated by integrating it across the absorption image map obtained from the transfer matrix method.

### Extraction of Refractive Index

The spectral response (from 200 to 1300 nm) of the transmittance and reflectance of a FA_0.8_Cs_0.2_Pb­(I_0.6_Br_0.4_)_3_ perovskite film is measured using an Agilent Cary 7000 Universal Measurement Spectrophotometer with the universal measurement accessory (UMA) at angles of 0, 8, 20, 30, 40, 50, and 60°, respectively, for both s- and p-polarization. The perovskite film is deposited on an ITO/MeO-2PACz to ensure consistent perovskite growth, as implemented in the photodetector device. We employ a code based on the transfer matrix method to extract the spectral response of the perovskite complex refractive index (*n* + i*k*) based on the Forouhi-Bloomer model^46^ using three oscillators by fitting the angular response of the reflectance and transmittance spectra. We implement a genetic algorithm to optimize the complex refractive index fitting, by including the thicknesses of the different layers, according to the experimental characterization in the structure as one of the fitting parameters.

### Fabrication of 1-Dimensional Photonic Crystal

1-Dimensional photonic crystals made of three pairs of alternating titanium dioxide (TiO_2_) and silicon dioxide (SiO_2_) layers were deposited on glass substrates. The thickness of each layer was TiO_2_ = (70 ± 2) nm and SiO_2_ = (90 ± 2) nm. The photonic crystal was deposited in an e-beam evaporation system with plasma source assistance (Syrus 710 Pro, Bühler Leybold Optics). The base pressure of the system was 2 × 10^–6^ mbar. TiO_2_ and SiO_2_ were deposited at 0.25 and 0.6 nm s^–1^ rates, respectively.

### ITO Sputtering

ITO electrodes were sputtered on glass substrates (for reference PD) and photonic crystal substrates (for cavity PD) with a home-built setup in the Class 10,000 clean room in the Electrical Engineering Division, Department of Engineering, University of Cambridge. A metal mask was used to pattern the ITO electrodes. An In_2_O_3_/SnO_2_ 90/10 wt % target was used for sputtering at an argon flow of 20 sccm, pressure of 5 mTorr, and power of 40 W. The rate of sputtering was 3.7 nm per minute, resulting in an ITO conductivity of 1800 S cm^–1^ measured with a 4-point-probe and an ITO thickness of 83 nm measured under AFM.

### Materials

Isopropyl alcohol (IPA, 99.5%), ethanol (EtOH), N,N-dimethylformamide (DMF, anhydrous, 99.8%), dimethyl sulfoxide (DMSO, anhydrous, 99.9%), chlorobenzene (CB, anhydrous, 99.8%), cesium bromide (CsBr, 99.999%), cesium iodide (CsI, 99.999%), and polyvinylpyrrolidone (PVP) were purchased from Sigma-Aldrich. Formamidinium bromide (FABr) and formamidinium iodide (FAI) were purchased from Greatcell Solar. Lead­(II) bromide (PbBr_2_, 99.999%), lead­(II) iodide (PbI_2_), and [2-(3,6-dimethoxy-9H-carbazol-9-yl)­ethyl]­phosphonic acid (MeO-2PACz) were purchased from TCI. Carbon-60 (C_60_) and bathocuproine (BCP) were purchased from Creaphys and Ossilla, respectively. Silver (Ag) was purchased from KJ Lesker. All chemicals were used without further purification.

### Preparation of FA_0.8_Cs_0.2_Pb­(I_0.6_Br_0.4_)_3_ Perovskite Precursor Solution

Perovskite precursor was prepared by dissolving FAI (0.48 mol), FABr (0.32 mol), CsI (0.12 mol), CsBr (0.08 mol), PbI_2_ (0.6 mol), and PbBr_2_ (0.4 mol) in DMF: DMSO at a ratio of 4:1. The dissolved precursor solution is then further diluted to different concentrations for thickness variation.

### Fabrication of Reference and Cavity Perovskite Photodetectors

Recycled substrates were sonicated in acetone 2 times for 10 min each. Both recycled and new ITO sputtered glass and photonic crystal substrates were then cleaned in detergent, deionized water, acetone, and isopropanol under ultrasonication for 10 min each, then treated with UV ozone for 15 min. The substrates were then transferred to a nitrogen-filled glovebox. MeO-2PACz (3 mg mL^–1^ in ethanol) was dropped on the substrate for 10 s before being spin-coated onto the substrate at 3000 rpm for 30 s and immediately annealed at 100 °C for 10 min. PVP (0.1 mg mL^–1^ in IPA) was spin-coated at 3000 rpm for 30 s and post-annealed at 100 °C for 5 min to improve wettability. The perovskite precursor was 2-step spin-coated at 2000 rpm for 10 s and then 6000 rpm for 40 s. During 10 s of the second spin step, 100 μL of chlorobenzene antisolvent was dripped onto the substrate, followed by a postannealing at 100 °C for 15 min. C_60_ (20 nm), BCP (8 nm), and Ag (100 nm) were then sequentially thermal evaporated on the perovskite film. The device area defined by the overlap area between ITO and the Ag electrode was 4.5 mm^2^.

### Film Thickness

The film thicknesses of the structure were assessed using a Bruker Dimension Icon AFM with a Bruker Scanasyst-Air cantilever running on peak force tapping mode. A scan was made across the depth of either a scratch made on the soft perovskite film with a razor blade or an IPA cleaned marker pen line drawn before ITO sputtering to reveal a clean edge without ITO. Data was analyzed with WSxM 5.0 software.^47^

### Reflectance

The cavity resonance (reflectance) was characterized using an Agilent Cary 7000 Universal Measurement Spectrophotometer with the universal measurement accessory (UMA). Due to the alignment limitation caused by the detector blocking the excitation beam path, the smallest reflectance angle that can be measured is 6°, where 0° is defined as perpendicular to the sample surface. The angle-resolved reflectance was measured by setting the sample angle at θ = 7.5, 15, 22.5, 30, 45, and 60° to the excitation beam, and the specular reflectance was collected.

### Photodetector EQE and Responsivity

The responsivity spectrum was measured with the Bentham PVE300 system across a spectral range of 300 to 750 nm with a step size of 2 and 5 nm. The system was equipped with a xenon-quartz tungsten halogen dual source, a single monochromator as light source, a chopper operating at a frequency of 600 Hz, and the device response was recovered with the Bentham 474 transformer, ultralow-noise preamplifier, and Bentham 496 lock-in amplifier. A silicon reference cell was used for the calibration. The angle-resolved responsivity was measured by placing the device on an adjustable angle plate (Thorlabs AP180) at θ = 7.5, 15, 22.5, 30, 45, and 60° to the excitation beam, calibrated with silicon reference cell placed at similar angles, device area of both reference cell and perovskite photodetector kept constant with a metal mask.

Responsivity is defined as the photocurrent generated per watt of optical signal and is wavelength-dependent. Responsivity, *R*, and EQE are given as a function of

$$\mathrm{EQE}=\frac{\mathrm{hc}}{q\lambda }R$$

where *h* is the Planck constant, *c* is the speed of light, *q* is a unit charge, and λ is the wavelength.

Specific detectivity is a standard figure of merit indicating the sensitivity of the photodetector to convert the input optical signal to a measurable value. Specific detectivity, D* (in Jones) can be calculated from responsivity, *R*, and *i*_noise_ (in AHz^–1/2^) with active area *A* as

$$D^{*}=\frac{R\sqrt{A}}{i_{noise}}=\frac{\sqrt{A}}{NEP}$$

where *A* is the device area. NEP is a standard metric used to quantify the sensitivity and minimum detectable power per square root of a photodetector.

### Photodetector Time and Frequency Response

The photodetector was excited by modulated light emitted from a laser (Osram PL450B or Thorlabs L638P150), where the laser was aligned a meter away over free space and driven by an Agilent 33250A Function Generator (square wave, 50% duty cycle at a certain frequency). The resulting photodetector response was measured by an oscilloscope (Tektronix DPO 3032) to investigate the rise and fall times. The electrical time response determines the time a photodetector takes to react to changes in input light intensity and is defined by the rise and fall times of the photocurrent, changing between 10% and 90% of its steady-state peak and ground values.

The frequency response of the photodetector was evaluated in a similar way to that for the response time measurement, using a vector network analyzer (Keysight FieldFox N9913A). The laser was biased by a source meter to the DC operating point; meanwhile, the −10 dBm radio frequency signal from the vector network analyzer was combined with the DC signal via a bias-tee. The received optical signal from the photodetector was then fed back into the vector network analyzer, hence the frequency response and bandwidth of the device can be directly obtained from S_21_. The modulation bandwidth refers to the frequency range over which the detector can effectively detect and respond to light without significant signal distortion. Consequently, photodetectors with a larger bandwidth enable a higher signal transmission capability. The threshold is commonly defined as the frequency range over which the optical power is reduced to half of its maximum.

### Photodetector *I–V*, Linear Dynamic Range, and Others

The *I–V* curve and dark current of photodetectors were measured using a Keithley 2450 Source Measure Unit. The linear dynamic range of the photodetector is measured separately for low and high incident light power.

LDR indicates a photodetector’s ability to accurately detect and respond to a wide range of light intensities without saturation or distortion. LDR is defined by

$$\mathrm{LDR}=20\times \log\left(\frac{I_{\max}}{I_{\min}}\right)$$

where *I*_max_ and *I*_min_ are the upper and lower current bounds of the linear response, respectively.

The low-power range was measured using a xenon-quartz tungsten lamp, and the lamp intensity was reduced through the optical density filters, and the resulting response was captured through the Bentham 474 transformer, ultralow-noise preamplifier, and Bentham 496 lock-in amplifier. The high-power range was excited using a Thorlabs L638P150 laser and optical density filters and measured using the Keithley 2450 Source Measure Unit.

The photocurrent stability of the photodetectors was measured using the Bentham PVE300 system at a fixed excitation of 635 nm.

The noise current of the spectrum analyzer and photodetectors was measured using the Moku:GO spectrum analyzer. Noise current can also be calculated from the sum of the shot noise and thermal noise.

Shot noise is defined as

$$i_{\mathrm{shot}}=\sqrt{2qI_{\mathrm{dark}}}$$

*i*_shot_ here has a unit of AHz^–1/2^ and *q* is a unit charge. The dark current, *I*_dark_, is estimated at 1.7 × 10^–7^ A cm^–2^, acquired from the current–voltage curve at −0.2 V bias, as shown in Figure 4b. The shot noise is thus estimated at 230 fA Hz^–1/2^.

Thermal noise (in AHz^–1/2^) is defined by

$$i_{\mathrm{thermal}}=\sqrt{\frac{4\mathrm{KT}}{R_{\mathrm{shunt}}}}$$

where *K*, *T*, and *R*_shunt_ are the Boltzmann constant, temperature in Kelvin, and shunt resistance of photodetector, respectively. The shunt resistance is calculated from the slope of the current density–voltage curve at a low voltage bias. The estimated shunt resistance is approximately 60 MΩ, giving a thermal noise of 16 fA Hz^–1/2^ at room temperature.

The theoretical noise is defined by

$$i_{noise}=\sqrt{{i_{shot}}^{2}+{i_{thermal}}^{2}}$$

### Cross-Section of Resonant Cavity-Enhanced Perovskite Photodetector

The TEM lamella cross-section was prepared using an FEI Helios Nanolab Dualbeam FIB/SEM instrument following standard protocols. The lamella was transferred with minimal air exposure to a Thermo Scientific Spectra 300 (S)­TEM operating at 300 kV and approximately 130 pA beam current. High-angle annular dark-field (HAADF) images were captured by using a Fischione detector with a camera length of 58 mm, a dwell time of 1 μs, and a spatial sampling of 1.5 nm per pixel for high-resolution data acquisition. Energy-dispersive X-ray spectroscopy (STEM-EDX) maps were acquired using four Super-X detectors with a dwell time of 50 ms, spatial sampling of 5 nm per pixel, and a spectral resolution of 10 eV per channel. Additionally, STEM-EDX spectrum images were obtained with a beam current of 150 pA, utilizing 40 frames, a convergence angle of 24 mrad, and a camera length of 58 mm. The STEM-EDX data were denoised using principal component analysis and processed using HyperSpy.^48^

## Supplementary Material



## Notes

- Details, presentation, and discussion of additional results relating to the optical modeling and optimization, further experimental results, and a comparison of the outcomes in this work with published counterparts (PDF)

## Notes

Details on the code that supports the findings of this study can be shared upon request. The data that support the findings of this study are openly available in Apollo—University of Cambridge Repository at https://doi.org/10.17863/CAM.119830.
