# Supplementary material for: Resonant Cavity Effect for Spectrally Tunable and Efficient Narrowband Perovskite Photodetectors
Source: ACS Photonics. 2025 Jul 21;12(8):4119–29. doi: 10.1021/acsphotonics.4c01942 (PMC12372166; doi:10.1021/acsphotonics.4c01942)
Supplement: Supplementary file 1 [file ph4c01942_si_001.pdf]

Resonant cavity effect for spectrally tunable and efficient narrowband perovskite photodetectors

Zher Ying Ooi,[1] Shenyu Nie,[1] Guadalupe Vega,[2,3] May Ching Lai,[4] Alberto Jiménez-Solano,[3] Chieh-Szu Huang,[1] Hao Wang,[5] Tianjun Liu,[6] Krzysztof Gałkowski,[6,7] Michał P. Nowak,[8] Piotr Nyga,[8] Qixiang Cheng,[5] Caterina Ducati,[4] Sol Carretero-Palacios,[9] Simon Kahmann,[1,10] Samuel D. Stranks,[1,6] Miguel Anaya[1,2]

1. Department of Chemical Engineering and Biotechnology, University of Cambridge, Cambridge, UK
2. Instituto de Ciencia de Materiales de Sevilla, Universidad de Sevilla–CSIC, Calle Américo Vespucio 49, Sevilla, 41092, Spain
3. Departamento de Física, Universidad de Córdoba, Edificio Einstein (C2), Campus de Rabanales, 14071, Córdoba, Spain
4. Department of Materials Science and Metallurgy, University of Cambridge, Cambridge, UK
5. Division of Electrical Engineering, Department of Engineering, University of Cambridge, Cambridge, UK
6. Cavendish Laboratory, University of Cambridge, Cambridge, UK
7. Department of Experimental Physics, Faculty of Fundamental Problems of Technology, Wroclaw University of Science and Technology, Wroclaw, Poland
8. Institute of Optoelectronics, Military University of Technology, Warsaw, Poland
9. Instituto de Ciencia de Materiales de Madrid, ICMM-CSIC, 28049 Madrid, Spain
10. Institute of Physics, Chemnitz University of Technology, Chemnitz, Germany

*sds65@cam.ac.uk, *anaya@us.es

Supporting Note 1 | Suppression of photodetection response at 400 nm

As shown in Figure S7, a second photodetection peak at 400 nm is observed, which falls outside the photonic crystal stopband. The EQE enhancement, determined by dividing the EQE of resonant cavity enhanced photodetectors with that of the reference at the same perovskite thickness, shows neither enhancement nor attenuation at 400 nm across all perovskite thicknesses. Conversely, within the photonic stopband, except at the resonance wavelength, the EQE is significantly attenuated. This indicates that the presence of the second photodetection peak at 400 nm, is dependent on the photonic stopband. While the experimental results in this work show the 400 nm second peak due to the availability of photonic crystal, simulations in Figure S8 suggest that a new photonic crystal design could eliminate this peak.

Supporting Note 2 | Resonant cavity-enhanced photodetectors from other absorber materials

Chen *et al.* utilized an AlGaAs/GaAs semiconductor with a metal-metal cavity for a resonant cavity-enhanced heterostructure.[1] They demonstrated wavelength selectivity at 850 nm with a narrowband response of 30 nm FWHM. Their delta-doped device achieved a six-fold enhancement using the resonant cavity, reaching 21.3% quantum efficiency and 0.15 A W^{-1} responsivity at a 10 V bias. With small device area of 40×40 um^2 and a minimized capacitance to 30 fF, their photodetector exhibited rise and fall times of less than 20 ps.

Casalino *et al.* reported a graphene-Silicon resonant cavity-enhanced Schottky photodetector operating at 1550 nm and achieving a responsivity of 0.25 A W^{-1} at a 10 V bias.[2] The calculated noise current, including both shot and thermal noise, is approximately 7.4 pA $Hz^{-1/2}$. From this, they determined a NEP of 3.5×10^{-10} W $Hz^{-1/2}$ and a specific detectivity, D^* of 5.1×10^7 Jones.

Siegmund *et al.* present a resonant cavity-enhanced organic photodetector featuring a 50 nm thick organic absorber blend within a metal-metal cavity. This design achieves a narrowband FWHM of 36 nm, an EQE exceeding 20% with up to 41-fold enhancement, and wavelength tunability from 810 to 1550 nm.[3] Additionally, it demonstrates a 50 nm wavelength shift at a 45° angle. The photodetector boasts a linear dynamic range of 108 dB and a shot noise current of 10^{-13} A $Hz^{-1/2}$.

Luo et al. reported a colloidal quantum dots photodetector integrated into a Fabry-Perot cavity, operating in the short-wave and mid-wave infrared range.[4] It achieved a responsivity of up to 1.6 A W^{-1} and a detectivity of 2×10^{11} Jones, using optical spacers range between 1.5 and 2.5 µm thick. This design showed a twofold enhancement compared to the reference.

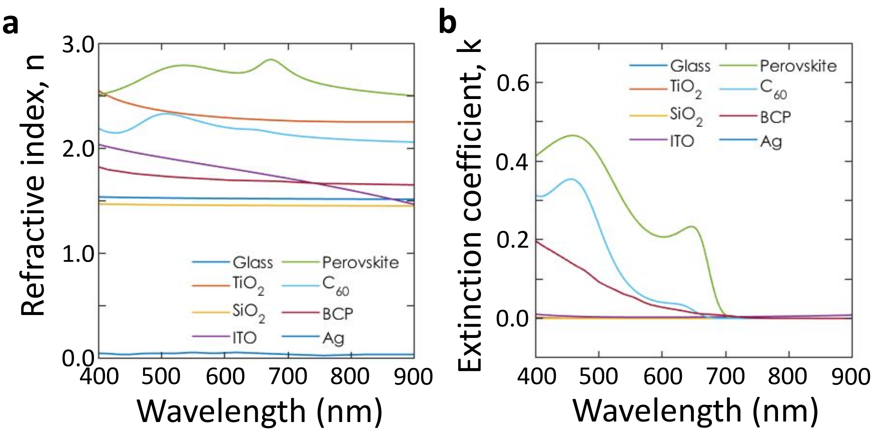

Figure S1. Optical constants of materials used in simulations. a, *Real, n, and* ***b,*** *imaginary, k, part of the complex refractive index of glass, TiO_2,*[5] *SiO_2,*[5] *ITO,*[6] *$FA_{0.8}Cs_{0.2}Pb(I_{0.6}Br_{0.4})_3$ perovskite, C_{60},*[7] *BCP*[8] *and Ag.*[9]

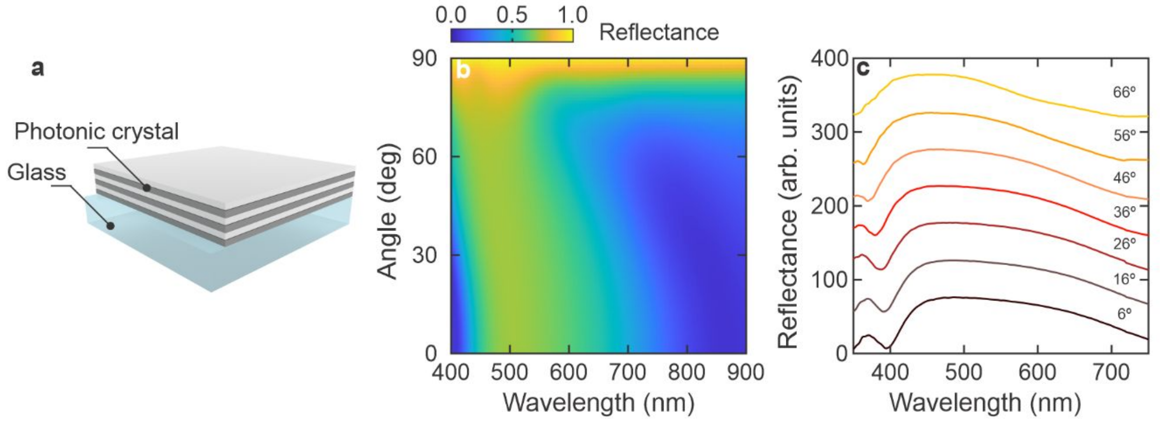

Figure S2. Photonic crystal. a, *Schematic of the photonic crystal structure comprised of 3-bilayers of TiO_2/SiO_2 on glass substrate.* ***b,*** *Simulated and* ***c,*** *experimental angle-dependent reflectance.*

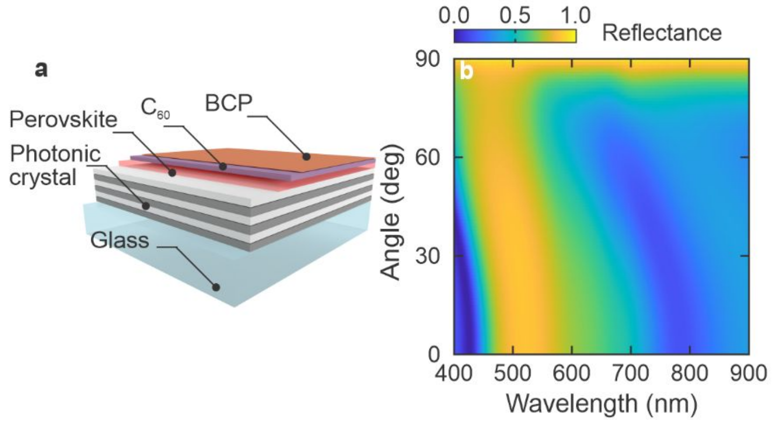

Figure S3. Photonic crystal with perovskite and transport layers. a, *Schematic of the photonic crystal structure comprised of 3-bilayers of TiO_2/SiO_2 on glass substrate, perovskite layer and C_{60}/BCP transport layers.* ***b,*** *Simulated reflectance of the structure.*

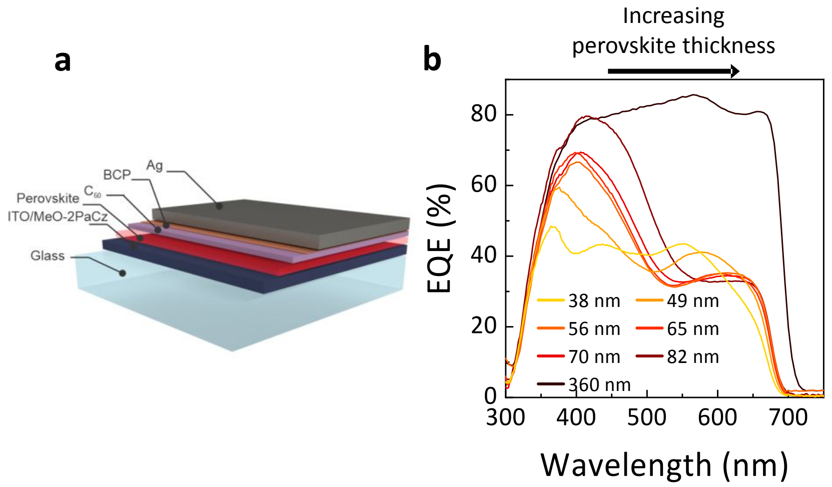

Figure S4. Reference perovskite photodetectors. a, *Schematic of reference perovskite photodetector.* ***b,*** *Measured EQE of reference perovskite photodetectors with perovskite thickness of 38 nm, 49 nm, 56 nm, 65 nm, 70 nm, 82 nm and 360 nm.*

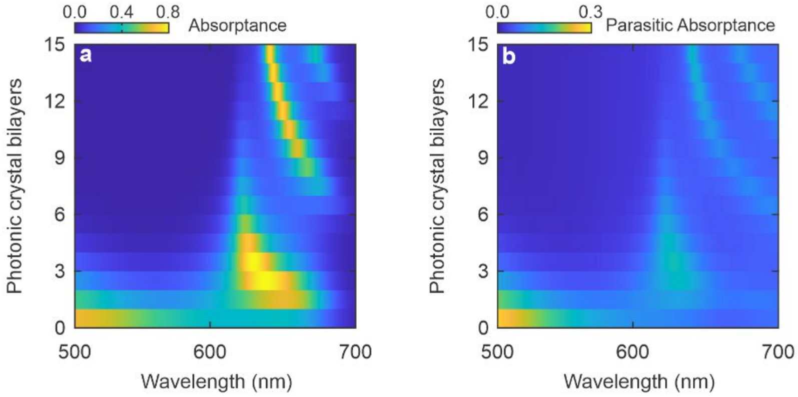

Figure S5. Simulation of various number of photonic crystal bilayers *for* ***(a)*** *absorption within the perovskite layer, and* ***(b)*** *parasitic absorption, which accounts for the total absorption within all other layers.*

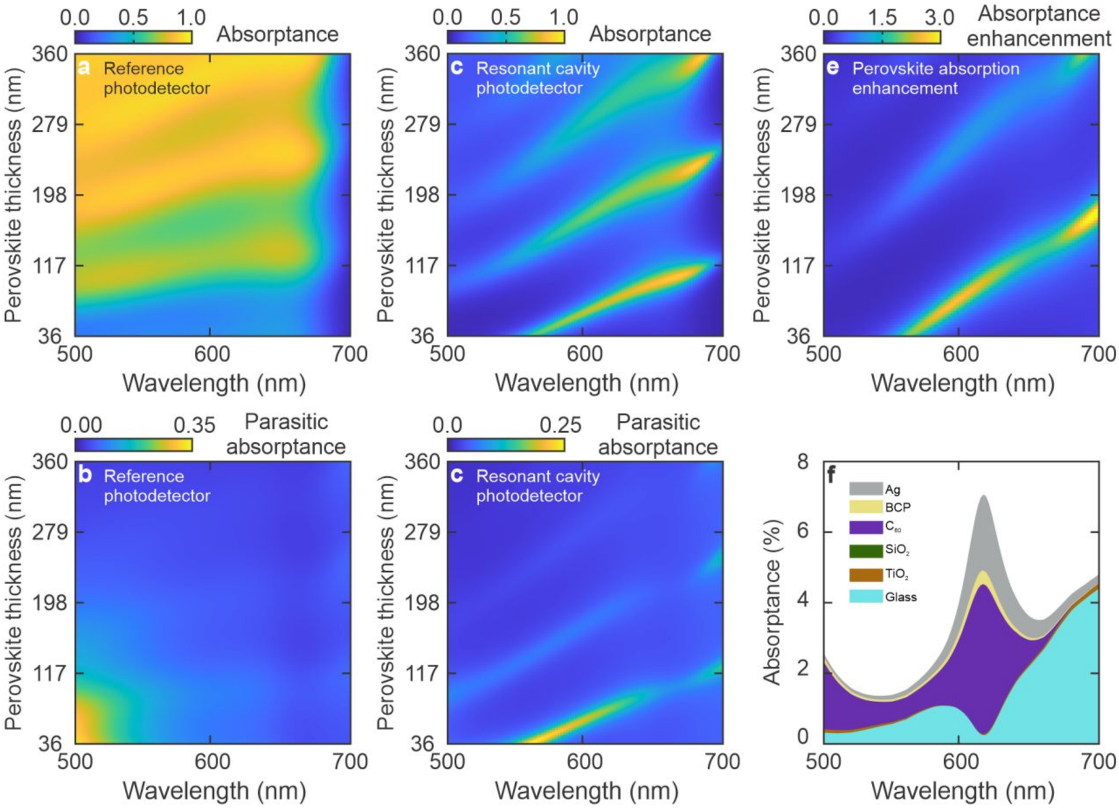

Figure S6. Simulated absorption across perovskite thickness. *Simulated total absorption within the perovskite layer in the device configuration of* ***(a)*** *reference photodetector and* ***(c)*** *resonant cavity photodetector. Simulated parasitic absorption, calculated as the total absorption across all other layers in the device configuration of* ***(b)*** *reference photodetector and* ***(d)*** *resonant cavity photodetector.* ***e,*** *Perovskite absorption enhancement calculated by the ratio of perovskite absorption within resonant cavity photodetector to that of the reference photodetector with the same perovskite thickness.* ***f,*** *(Continued from Figure 1) Absorption in other layers including glass,* TiO_2*,* SiO_2*,* C_{60}*, BCP and Ag.*

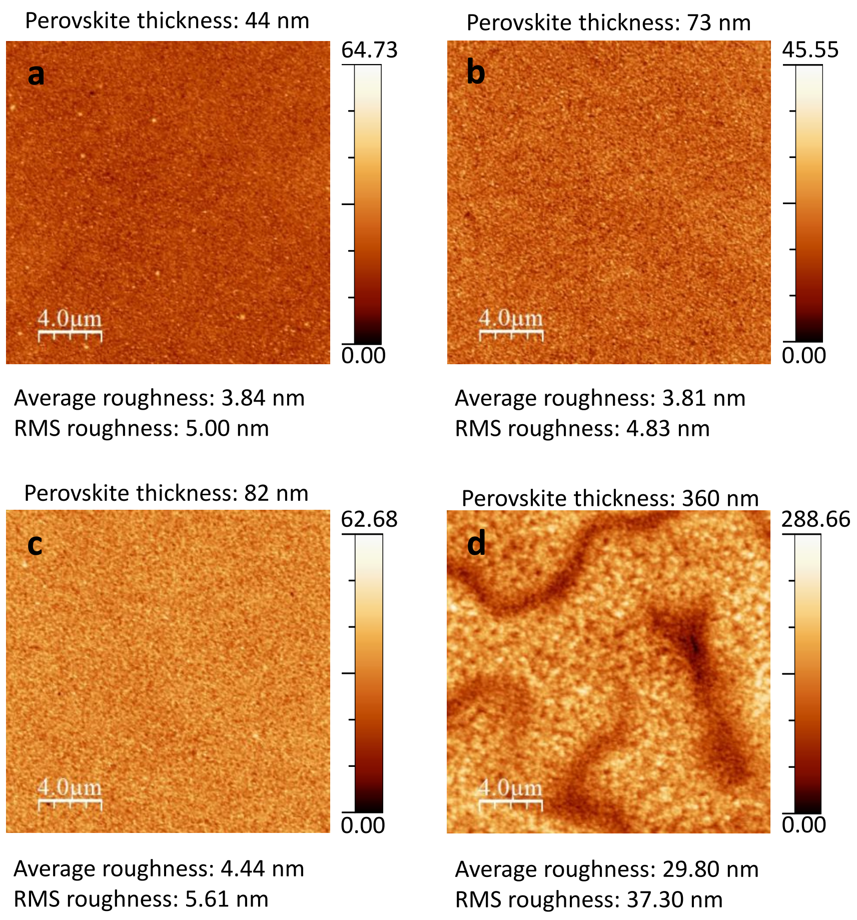

Figure S7. Perovskite roughness. *Perovskite roughness measured with an atomic force microscope for perovskite film with thickness of* ***a,*** *44 nm,* ***b,*** *73 nm,* ***c,*** *82 nm and* ***d,*** *360 nm.*

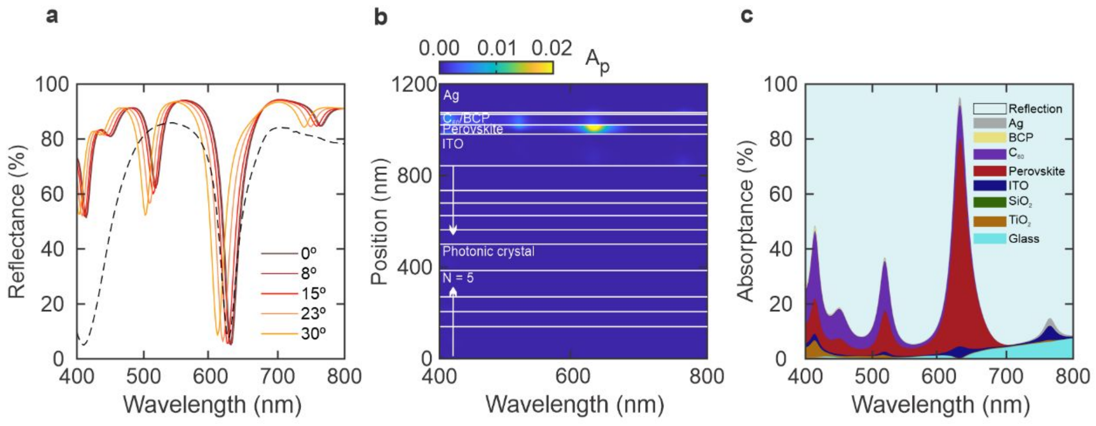

Figure S8. Simulated resonant cavity perovskite photodetector with advanced photonic crystal to remove 400 nm peak. *The advanced photonic crystal consists of 5 non-periodic bilayers.* ***a,*** *Simulated reflectance from 0° to 30°. Dashed line represents measured reflectance from Figure S9a with 82 nm thick perovskite.* ***b,*** *Simulated absorption across the cross-section of device stack across the spectrum wavelength.* ***c,*** *Simulated integrated absorption within the layers of the structure and the resulting reflection from the device stack.*

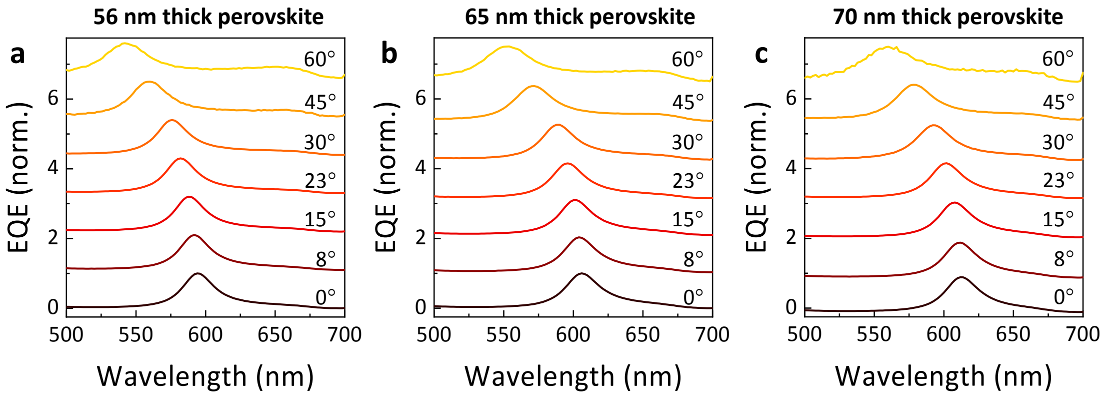

Figure S9. Angle dependent cavity resonance. *Measured angle-dependent EQE of resonant cavity photodetector with perovskite thickness of **(a)** 56 nm, **(b)** 65 nm and **(c)** 70 nm.*

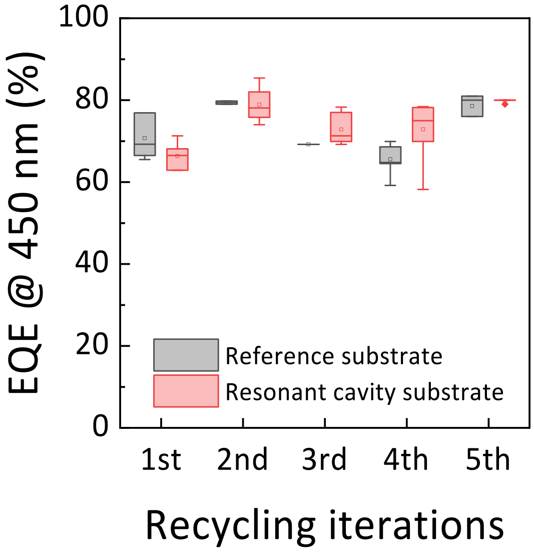

Figure S10. Recycling substrates. *The substrates for both reference and resonant cavity photodetectors were recycled with the same iterations to ensure consistency. The un-encapsulated photodetector devices were washed in acetone under sonication for 2 cycles before going through the standard substrate cleaning procedures (see Method). The EQE of each device measured were recorded at 450 nm because this wavelength shows minimum changes with perovskite thickness and was not dependent on the resonant cavity enhancement and photonic stopband attenuation. As optimisation was in progress while doing the recycling iterations, the EQE fluctuates slightly due to sample-to-sample variation.*

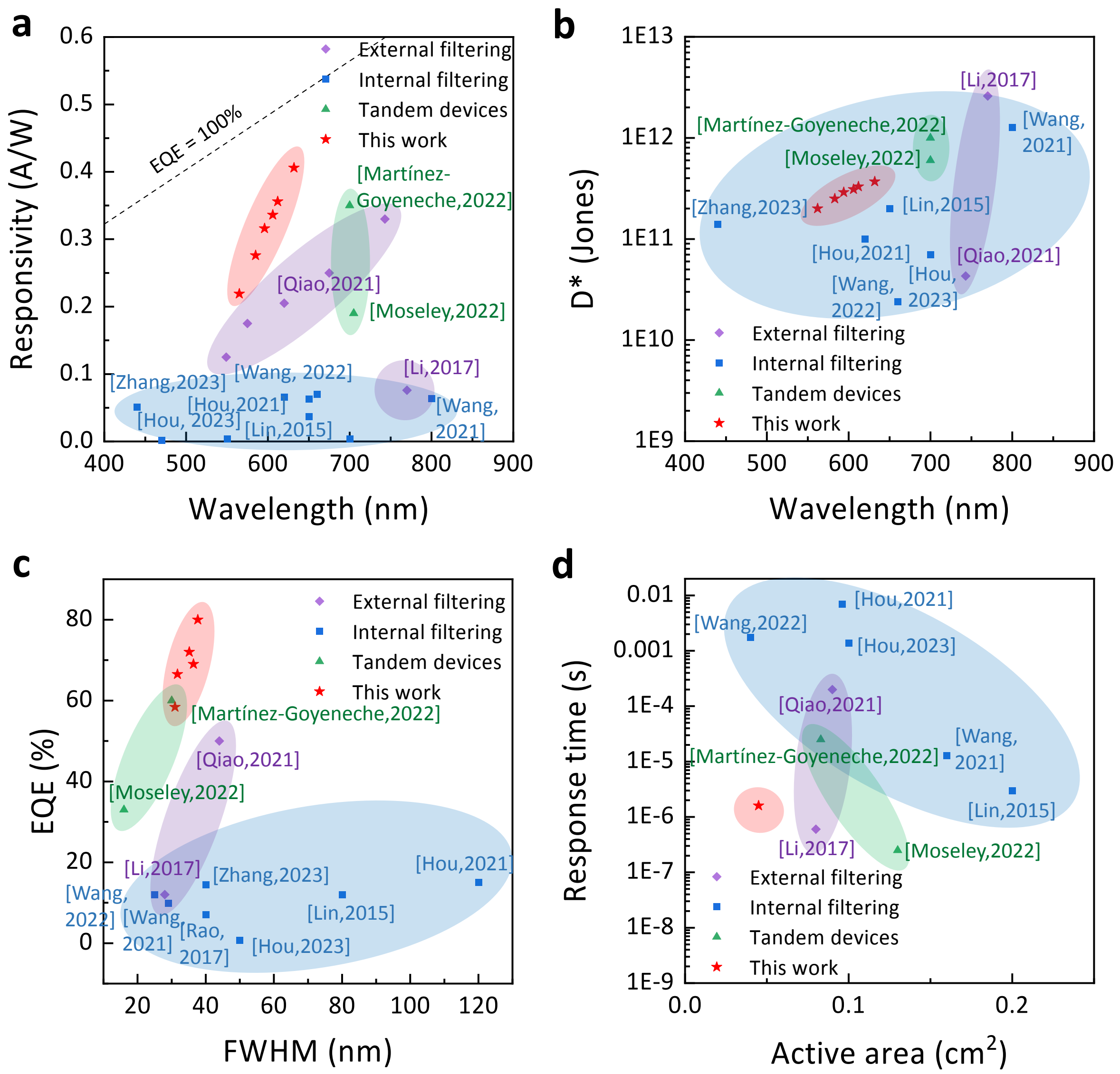

Figure S11. Performance of reported perovskite narrowband photodetectors. ***a)*** *Responsivity across wavelength,* ***b)*** *specific detectivity across wavelength, calculated assuming I_{dark}=1.7 x 10^{-7} A cm^{-2} and R_{shunt}=60 MΩ for all devices as per Figure 4b,* ***c)*** *EQE vs FWHM, and* ***d)*** *response time over device active area comparisons of reported perovskite narrowband photodetectors achieved from external filtering,[10,11] internal filtering through charge-collection narrowing,[12–18] tandem devices,[19,20] and this work. The details of the reported work are listed in Table S1.*

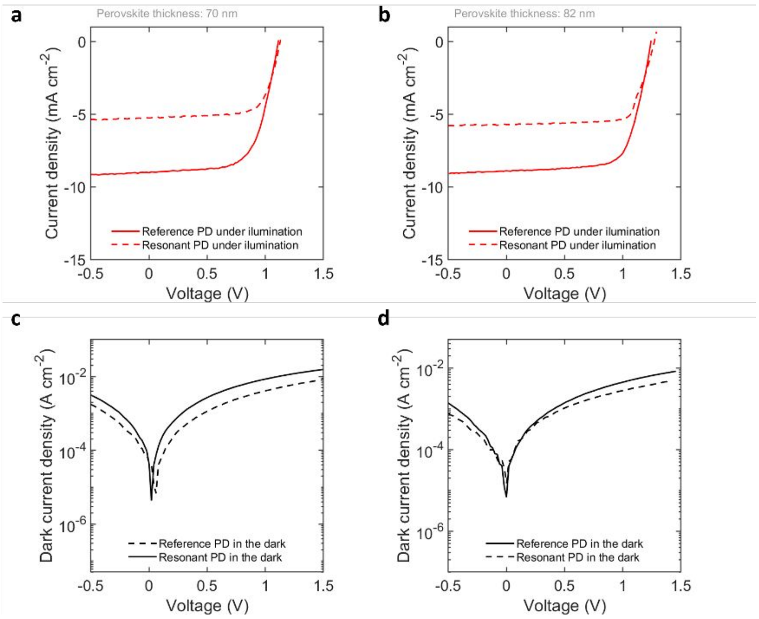

Figure S12. Current density-voltage curves, *measured in the dark and under AM1.5 illumination, are shown for photodetectors with perovskite thicknesses of 70 nm (panels a and c, respectively) and 82 nm (panels b and d, respectively). These measurements were acquired three months after keeping the devices from Figure 4b stored in a* N_2*-filled glovebox.*

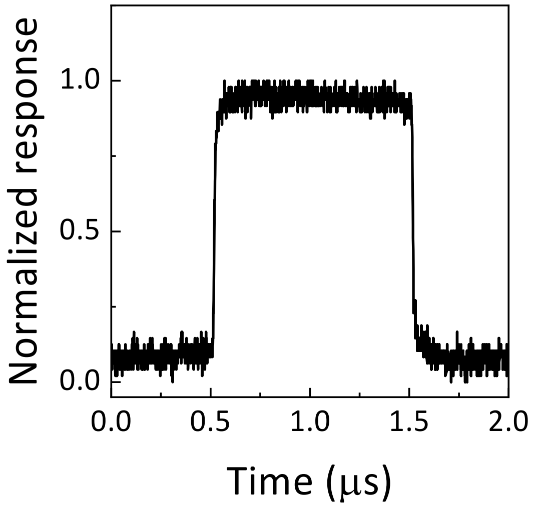

Figure S13. Instrument response of measurement setup. *The instrument response of measurement setup is validated with a silicon avalanche photodetector (Hamamatsu Si APD S12023) with faster response than the perovskite photodetector in this work. The rise and fall times of the silicon photodetector are both 0.03 μs, which are 10 times faster than the values shown in Figure 4d, thus the measurement is valid.*

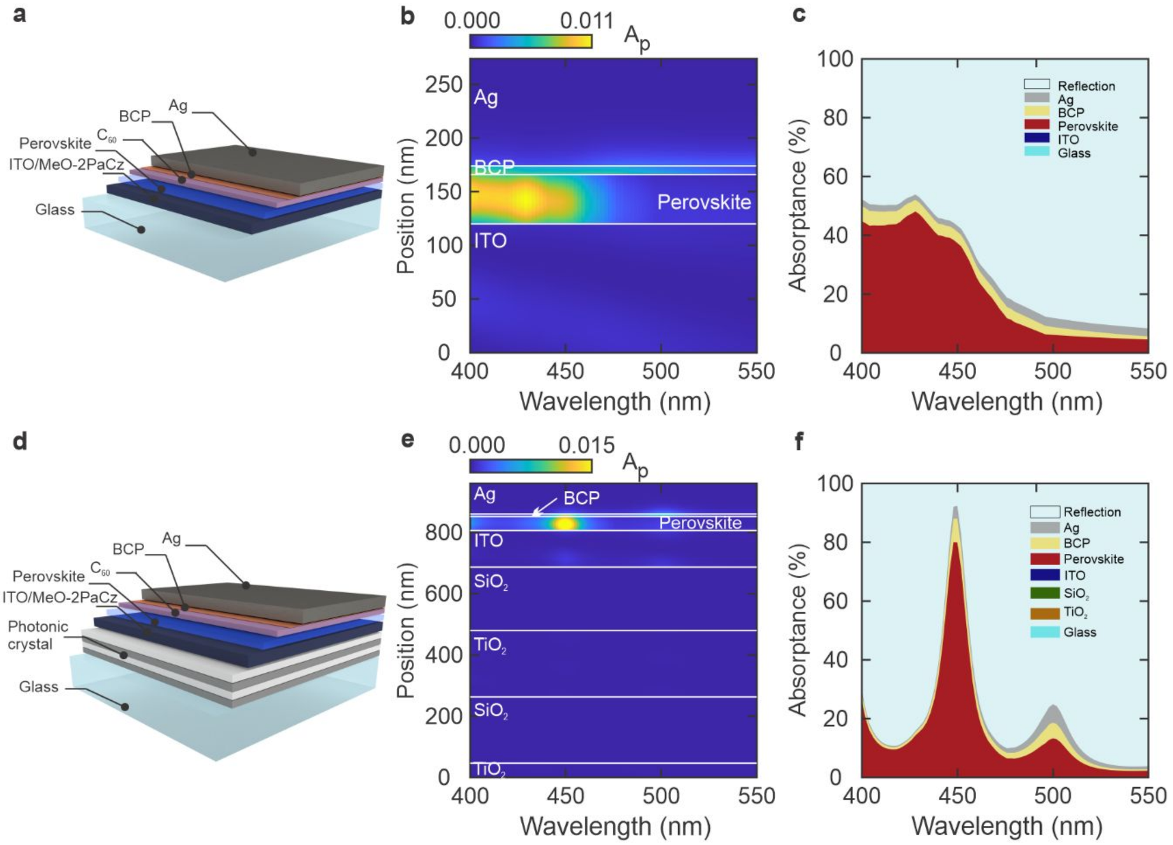

Figure S14 | Simulated optimised structure for blue photodetection. *Schematic of simulated* ***a,*** *reference and* ***d,*** *resonant cavity perovskite photodetector structures. Simulated absorption across the cross-section of reference device stack across the spectrum wavelength and simulated ratio of integrated absorption within each layer and the resulting reflection from the whole* ***(b,c)*** *reference and* ***(e,f)*** *resonant cavity perovskite photodetectors. Refractive index for blue perovskite acquired from reference.*[21] *C_{60} is not added in both structures because the bandgap of C_{60} is not suitable for large bandgap perovskite photovoltaics and photodetectors, thus an alternative structure is required.*[22,23]

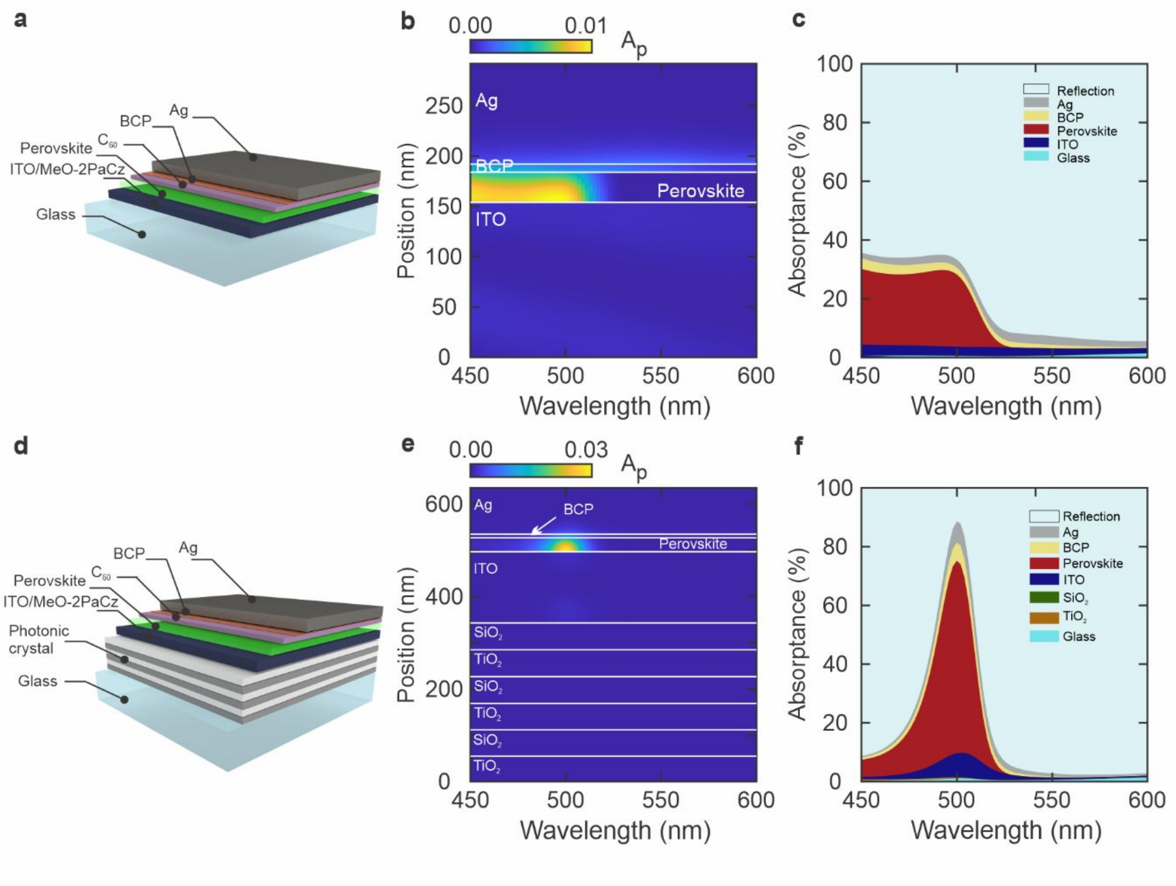

Figure S15 | Simulated optimised structure for green photodetection. *Schematic of simulated* ***a,*** *reference and* ***d,*** *resonant cavity perovskite photodetector structures. Simulated absorption across the cross-section of reference device stack across the spectrum wavelength and simulated ratio of integrated absorption within each layer and the resulting reflection from the whole* ***(b,c)*** *reference and* ***(e,f)*** *resonant cavity perovskite photodetectors. Refractive index for green perovskite acquired from reference.*[5] *C_{60} is not added in both structures because the bandgap of C_{60} is not suitable for large bandgap perovskite photovoltaics and photodetectors, thus an alternative structure is required.*[22,23]

Table S1 | Narrowband perovskite photodiodes with no/small bias voltage

Citation	Type	Perovskite thickness	Narrowband spectrum		Device performance				Response		
			Peak	FWHM	Bias	EQE	Responsivity	Detectivity	Time *	Speed	Active area
Li et al, 2017 [10]	Filter using perovskite film	20 µm	770 nm	28 nm	0 V	12 %	0.076 A/W	2.6×10^{12} Jones	600 ns	-	0.08 cm^2
Qiao et al, 2021 [11]	Filter using perovskite film	1 µm	743 nm	44 nm	0 V	50 %	0.33 A/W	4.3×10^{10} Jones	200 µs	-	0.09 cm^2
Lin et al., 2015 [15]	CCN of perovskite film doped with organics	500 nm	650 nm	80 nm	-0.5 V	12 %	0.063 A/W	2×10^{11} Jones	3 µs	297 kHz	0.2 cm^2
Rao et l., 2017 [18]	CCN of perovskite single crystal	400 µm	650 nm	40 nm	-1 V	7 %	0.037 A/W	-	350 µs	7 kHz	120 cm^2
Hou et al, 2021 [14]	CCN of perovskite thin film	2 µm	620 nm	120 nm	0 V	15 %	0.066 A/W	1×10^{11} Jones	6.9 ms	12 kHz	0.096 cm^2
Wang et al., 2021 [13]	CCN of perovskite thin film	25.1 µm	800 nm	29 nm	0 V	9.9 %	0.064 A/W	1.27×10^{12} Jones	12.7 µs	-	0.16 cm^2
Wang et al., 2022 [17]	CCN of perovskite thin film	3.9 µm	660 nm	25 nm	-0.3 V	12 %	0.07 A/W	2.4×10^{10} Jones	1.76 ms	252 Hz	0.04 cm^2
Hou et al, 2023 [16]	CCN of perovskite thin film	1.7 µm	700 nm	50 nm	0 V	0.7 %	0.004 A/W	6×10^{10} Jones	1.37 ms	17.8 kHz	0.1 cm^2
Zhang et al., 2023 [12]	CCN of perovskite thin film	300 µm	440 nm	40 nm	0.6 V	14.4 %	0.051 A/W	1.4×10^{11} Jones	920 µs	-	-
Moseley et al., 2022 [24]	Tandem device	650 nm	705 nm	16 nm	0.5 V	33 %	0.19 A/W	6×10^{11} Jones	250 ns	1.8 MHz	0.13 cm^2
Martínez-Goyeneche et al., 2022 [20]	Tandem device	600 nm	700 nm	30 nm	-1 V	60 %	0.35 A/W	1×10^{12} Jones	25 µs	-	0.083 cm^2
**This work**	**Resonant enhanced cavity**	**82 nm**	**630 nm**	**38 nm**	**0 V**	**80 %**	**0.41 A/W**	**3.7×10^{11} Jones**	**0.92 µs**	**540 kHz**	**0.045 cm^2**

* The longer time is shown for either rise or fall times.

Table S2 | Broadband perovskite photodetectors

Year	Type of perovskite	Perovskite thickness	Broadband spectrum	Device performance				Response		
				Bias	EQE	Responsivity	Detectivity	Time *	Speed	Active area
Dou et al., 2014 [25]	$CH_3NH_3PbI_{3-x}Cl_x$ film	~ 300nm	300 – 800 nm	0 V	80 %	-	-	180 ns	2.9 MHz	0.01 cm^2
Lin et al., 2015 [26]	$CH_3NH_3PbI_3$ film	-	300 – 800 nm	0 V	70 %	-	3.1 x 10^{12} Jones	1.7 µs	500 kHz	0.2 cm^2
Bao et al., 2018 [27]	$CsPbI_xBr_{3-x}$ film	300 nm	400 – 580 nm	-0.3 V	57 %	0.28 A/W	9.7 x 10^{12} Jones	20 ns	2 MHz	0.01 cm^2
Zhang et al., 2019 [28]	$Cs_3Cu_2I_5$ single crystal	1.5 mm	< 405 nm	1 V	0.3 %	0.0649 A/W	6.9 x 10^{11} Jones	50 ms	-	0.0625 cm^2
Bao et al., 2020 [29]	$FAPbI_3$ film	50 nm	350 – 800 nm	0 – 1 V	25 %	-	5.3 x 10^{12} Jones	6.8 ns	65 MHz	0.001 cm^2
Wang et al., 2020 [30]	$CsPbBr_3$ quantum dots	-	450 nm	1 V	-	0.14 A/W	7 x 10^{11} Jones	38 ms	-	0.11 cm^2
Wu et al., 2021 [31]	$CsPbBr_3$ film	150 nm	350 – 510 nm	5 V	-	3.15 A/W	3.9 x 10^{12} Jones	8 ms	300 Hz	-
Zhang et al., 2022 [32]	$MAPbI_3$ film	~ 500 nm	300 – 800 nm	0 V	88 %	0.43 A/W	-	25 µs	-	0.16 cm^2
Moseley et al., 2023 [33]	$Cs_{0.05}FA_{0.79}MA_{0.16}Pb(I_{0.84}Br_{0.16})_3$	-	350 – 750 nm	-1 V	79 %	-	1.4 x 10^{11} Jones	2 µs	190 kHz	0.1 cm^2
Lai et al., 2024 [34]	$CsPbI_2Br$ 2D/3D heterojunction	-	300 – 700 nm	0 V	90 %	0.41 A/W	1.0 x 10^{13} Jones	2 µs	-	0.07 cm^2

* The longer time is shown for either rise or fall times.

References

(1) Chen, X. *et al.* Optical and electrical characterization of GaAs-based high-speed and high-sensitivity delta-doped resonant cavity-enhanced HMSM photodetector. *IEEE Trans. Electron Devices*, **2005**, 52, 454–464.

(2) Casalino, M. *et al.* Vertically Illuminated, Resonant Cavity Enhanced, Graphene–Silicon Schottky Photodetectors. *ACS Nano*, **2017**, 11, 10955–10963.

(3) Siegmund, B. *et al.* Organic narrowband near-infrared photodetectors based on intermolecular charge-transfer absorption. *Nat. Commun.*, **2017**, 8, 15421.

(4) Luo, Y., Zhang, S., Tang, X. & Chen, M. Resonant cavity-enhanced colloidal quantum-dot dual-band infrared photodetectors. *J. Mater. Chem. C*, **2022**, 10, 8218–8225.

(5) Ooi, Z. Y. *et al.* Strong angular and spectral narrowing of electroluminescence in an integrated Tamm-plasmon-driven halide perovskite LED. *Nat. Commun.*, **2024**, 15, 5802.

(6) König, T. A. F. *et al.* Electrically Tunable Plasmonic Behavior of Nanocube–Polymer Nanomaterials Induced by a Redox-Active Electrochromic Polymer. *ACS Nano*, **2014**, 8, 6182–6192.

(7) Ávila, J. *et al.* High voltage vacuum-deposited CH3NH3PbI3–CH3NH3PbI3 tandem solar cells. *Energy Environ. Sci.*, **2018**, 11, 3292–3297.

(8) Hossain, M. I. *et al.* Electrical and Optical Properties of Nickel-Oxide Films for Efficient Perovskite Solar Cells. *Small Methods*, **2020**, 4, 2000454.

(9) Johnson, P. B. & Christy, R. W. Optical Constants of the Noble Metals. *Phys. Rev. B*, **1972**, 6, 4370–4379..

(10) Li, L. *et al.* Self-Filtered Narrowband Perovskite Photodetectors with Ultrafast and Tuned Spectral Response. *Adv. Opt. Mater.*, **2017**, 5, 1700672.

(11) Qiao, S., Liu, Y., Liu, J., Fu, G. & Wang, S. High-Responsivity, Fast, and Self-Powered Narrowband Perovskite Heterojunction Photodetectors with a Tunable Response Range in the Visible and Near-Infrared Region. *ACS Appl. Mater. Interfaces*, **2021**, 13, 34625–34636.

(12) Zhang, Z.-Y. *et al.* A Narrowband Perovskite Photodetector for Blue Light Hazard Detection. *IEEE Trans. Electron Devices*, **2023**, 70, 5146–5150.

(13) Wang, J. *et al.* Self-Driven Perovskite Narrowband Photodetectors with Tunable Spectral Responses. *Adv. Mater.*, **2021**, 33, 2005557.

(14) Hou, Y. *et al.* Self-Powered Red/UV Narrowband Photodetector by Unbalanced Charge Carrier Transport Strategy. *Adv. Funct. Mater.*, **2021**, 31, 2007016.

(15) Lin, Q., Armin, A., Burn, P. L. & Meredith, P. Filterless narrowband visible photodetectors. *Nat. Photonics*, **2015**, 9, 687–694..

(16) Hou, Y. *et al.* Retina-inspired narrowband perovskite sensor array for panchromatic imaging. *Sci. Adv.*, 2023, 9, eade2338.

(17) Wang, X. *et al.* Spray-Coating Thick Films of All-Inorganic Halide Perovskites for Filterless Narrowband Photodetectors. *ACS Appl. Mater. Interfaces*, **2022**, 14, 24583–24591.

(18) Rao, H., Li, W.-G., Chen, B.-X., Kuang, D.-B. & Su, C.-Y. In Situ Growth of 120 cm2 CH3NH3PbBr3 Perovskite Crystal Film on FTO Glass for Narrowband-Photodetectors. *Adv. Mater.*, **2017**, 29, 1602639.

(19) Moseley, O. D. I. *et al.* Tunable Multiband Halide Perovskite Tandem Photodetectors with Switchable Response. *ACS Photonics*, **2022**, 9, 3958–3966.

(20) Martínez-Goyeneche, L. *et al.* Narrowband Monolithic Perovskite–Perovskite Tandem Photodetectors. *Adv. Opt. Mater.*, **2022**, 10, 2201047.

(21) Chen, Z. *et al. Utilization of Trapped Optical Modes for White Perovskite Light-Emitting Diodes with Efficiency over 12%, **2021**, 5, 456-466.*

(22) Lim, J. W. *et al.* Unprecedentedly high indoor performance (efficiency > 34 %) of perovskite photovoltaics with controlled bromine doping. *Nano Energy*, **2020**, 75, 104984.

(23) Hou, S. *et al.* Efficient CsPbBr3 Perovskite Solar Cells with Storage Stability > 340 Days. *Energies*, **2022**, 15, 7740.

(24) Moseley, O. D. I. *et al.* Tunable Multiband Halide Perovskite Tandem Photodetectors with Switchable Response. *ACS Photonics*, **2022**, 9, 3958–3966.

(25) Dou, L. *et al.* Solution-processed hybrid perovskite photodetectors with high detectivity. *Nat. Commun.*, **2014**, 5, 5404.

(26) Lin, Q., Armin, A., Lyons, D. M., Burn, P. L. & Meredith, P. Low Noise, IR-Blind Organohalide Perovskite Photodiodes for Visible Light Detection and Imaging. *Adv. Mater.*, **2015**, 27, 2060–2064.

(27) Bao, C. *et al.* High Performance and Stable All-Inorganic Metal Halide Perovskite-Based Photodetectors for Optical Communication Applications. *Adv. Mater.*, **2018**, 30, 1803422.

(28) Zhang, Z.-X. *et al.* Sensitive Deep Ultraviolet Photodetector and Image Sensor Composed of Inorganic Lead-Free Cs3Cu2I5 Perovskite with Wide Bandgap. *J. Phys. Chem. Lett.*, **2019**, 10, 5343–5350.

(29) Bao, C. *et al.* Bidirectional optical signal transmission between two identical devices using perovskite diodes. *Nat. Electron.*, **2020**, 3, 156–164.

(30) Wang, H., Zhang, P. & Zang, Z. High performance CsPbBr3 quantum dots photodetectors by using zinc oxide nanorods arrays as an electron-transport layer. *Appl. Phys. Lett.*, **2020**, 116, 162103.

(31) Wu, W. *et al.* Ultrathin and Conformable Lead Halide Perovskite Photodetector Arrays for Potential Application in Retina-Like Vision Sensing. *Adv. Mater.* , **2021**, 33, 2006006.

(32) Zhang, X. *et al.* Rubidium Iodide-Doped Spiro-OMeTAD as a Hole-Transporting Material for Efficient Perovskite Photodetectors. *J. Phys. Chem. C*, **2022**, 126, 9528–9540.

(33) Moseley, O. D. I., Roose, B., Zelewski, S. J. & Stranks, S. D. Identification and Mitigation of Transient Phenomena That Complicate the Characterization of Halide Perovskite Photodetectors. *ACS Appl. Energy Mater.*, **2023**, 6, 10233–10242.

(34) Lai, L., Liu, G., Zhou, Y., He, X. & Ma, Y. Modulating Dimensionality of 2D Perovskite Layers for Efficient and Stable 2D/3D Perovskite Photodetectors. *ACS Appl. Mater. Interfaces*, **2024**, 16, 19849–19857.
